# A new era of political-technical synergy in Africa’s cholera response

**DOI:** 10.4102/jphia.v16i1.1646

**Published:** 2025-10-10

**Authors:** Ngashi Ngongo, Nebiyu Dereje, Roma Chilengi, Jean Kaseya

**Affiliations:** 1 Africa Centres for Disease Control and Prevention, Addis Ababa, Ethiopia; 2 Zambia National Public Health Institute (ZNPHI), Lusaka, Zambia

In a historic demonstration of political resolve, African leaders have launched a bold and coordinated response to the escalating cholera outbreaks in multiple countries.^1,2^ Spearheaded by the Africa Centres for Disease Control and Prevention (Africa CDC), the new cholera response plan marks a paradigm shift in public health governance – placing Heads of State at the forefront of epidemic control and leveraging lessons from recent outbreaks, including mpox, to drive a multisectoral and integrated approach. This shift was driven by changes in the epidemiological situation of the cholera outbreak, which has expanded in scale and severity, affecting multiple countries across the continent.

Between 01 January 2025 and 03 August 2025, a total of 206 789 cases and 4330 deaths of cholera have been reported from 23 member states: Angola (27 666 cases; 773 deaths), Burundi (408 cases; 0 deaths), Chad (77 cases; 4 deaths), Congo Republic (214 cases; 21 deaths), Comoros (40 cases; 0 deaths), Côte d’Ivoire (109 cases; 7 deaths), Democratic Republic of the Congo (DRC) (40 487 cases; 1102 deaths), Ethiopia (5755 cases; 49 deaths), Ghana (2780 cases; 14 deaths), Kenya (425 cases; 20 deaths), Malawi (91 cases; 3 deaths), Mozambique (4167 cases; 43 deaths), Namibia (17 cases; 1 deaths), Nigeria (2124 cases; 68 deaths), Rwanda (308 cases; 0 deaths), Somalia (6550 cases; 9 deaths), South Sudan (67 064 cases; 1142 deaths), Sudan (43 048 cases; 989 deaths), Tanzania (3892 cases; 40 deaths), Togo (165 cases; 4 deaths), Uganda (99 cases; 1 deaths), Zambia (483 cases; 9 deaths) and Zimbabwe (601 cases; 23 deaths).^3^ The most severely affected countries – Angola, the DRC, Sudan and South Sudan – face compounding challenges of insecurity, fragile health systems and limited access to clean water and sanitation.

## Political leadership as a game changer

Recognising the urgency, twenty African Union (AU) Member States convened in June 2025 for a high-level meeting led by H.E. Hakainde Hichilema, President of Zambia and AU Champion on Cholera. The meeting brought together ten Heads of State and Vice-Presidents, alongside ministers of health, finance and water and sanitation, to forge a united front against cholera. This resulted in a call to action to accelerate the response efforts and end cholera by 2030.^1^

The direct engagement of African Heads of State in the cholera response marks a decisive game changer for public health in Africa. For too long, epidemic responses have remained confined within the realm of technical agencies, often isolated from the political power needed to drive large-scale change. This new paradigm brings political authority and technical expertise into strategic alignment. It acknowledges that achieving lasting epidemic control and health security demands more than technical solutions; it requires unwavering political will, increased domestic investment and bold cross-sectoral leadership. With cholera’s root causes spanning water and sanitation, health systems, education and infrastructure, the sustained leadership of Presidents is not just beneficial – it is indispensable. Their involvement unlocks the coordinated, high-level action needed to fast-track progress and realise the continent’s vision of eliminating cholera by 2030.^4,5^

Following the launch of the continental Call to Action on cholera, H.E. Hakainde Hichilema, President of Zambia and African Union Champion on Cholera, is set to intensify his leadership by conducting high-level visits to the most affected countries. In a powerful show of peer engagement, President Hichilema will meet directly with fellow Heads of State to champion the establishment of Presidential Cholera Task Forces, advocate for increased domestic financing, advocate for greater domestic resource allocation and elevate the role of the water, sanitation and hygiene (WASH) sector as a central pillar in the cholera response. Supported by the Africa Centers for Disease Control-World Health Organization (CDC-WHO) led Incident Management Support Team (IMST), these visits will also serve to assess national response capacities and accelerate political accountability. This initiative marks a pivotal shift – anchoring cholera control at the highest political level and transforming the landscape of epidemic response in Africa.

The cholera response is strategically building on the lessons learned from the successful deployment of the mpox IMST, which proved instrumental in coordinating multi-country action during the 2024 Public Health Emergency of Continental Security.^6^ The mpox IMST demonstrated the power of rapid coordination, evidence-based interventions and community-centred approaches, setting a precedent for agile and integrated epidemic response in Africa. Drawing from this experience, the IMST model is now being scaled up to address multiple concurrent health threats, including cholera, with a deliberate focus on elevating political leadership. Unlike previous models that were largely technocratic, this next-generation IMST approach actively engages Heads of State and political decision-makers, ensuring high-level commitment, cross-sectoral coordination and sustained domestic investment. By institutionalising this integrated model, Africa CDC is forging a new path for resilient, leadership-driven epidemic response in a context of constrained resources and growing public health threats.^6,7,8^
